# Are attitudes toward peace and war the two sides of the same coin? Evidence to the contrary from a French validation of the Attitudes Toward Peace and War Scale

**DOI:** 10.1371/journal.pone.0184001

**Published:** 2017-09-11

**Authors:** Nicolas Van der Linden, Christophe Leys, Olivier Klein, Pierre Bouchat

**Affiliations:** 1 Center for Social and Cultural Psychology, Université Libre de Bruxelles, Brussels, Belgium; 2 Service d’Analyse des Données, Université Libre de Bruxelles, Brussels, Belgium; UNITED KINGDOM

## Abstract

Bizumic et al. (2013) have recently shown that attitudes towards peace and war reflect two distinct constructs rather than two poles of a single dimension. We present an attempt at validating the French version of their 16-item Attitudes toward Peace and War Scale (APWS) on five distinct (mainly Belgian) French-speaking samples (total N = 808). Confirmatory factor and criterion validity analyses confirmed that attitudes toward peace and war, although negatively related, are distinct in terms of their antecedents and consequences. On the one hand, antecedents of attitudes toward peace included egalitarian ideological beliefs and empathic concern for others, and consequences included intentions to engage in pro-peace behaviors. On the other hand, antecedents of attitudes toward war included national identification and authoritarian ideological beliefs, and consequences included intentions to engage in pro-war behaviors. Furthermore, both attitudes toward peace and war were, respectively, negatively and positively related to (a right-wing) political orientation. Unexpectedly however, attitudes toward war were positively related to nonegalitarian ideological beliefs and were not related to personal distress. Scores on the translated scale were unrelated to socially desirable responding. In terms of known-groups validity, men had, respectively, more and less positive attitudes toward war and peace than women. Finally, based on exploratory factor analyses, the inclusion of some items for the factorial structure of the measure is questioned and a shortened form of the measure is validated. Overall, these findings are in line with Bizumic et al. and suggest that attitudes toward peace and war also reflect two distinct constructs in a French-speaking population.

## Introduction

There is a paradox here. How can we honour those who died if we do not honour the cause for which they gave their lives? [1]

In view of the preparations of the commemorations marking the centenary of the First World War, resolutions have been drafted and experts have been solicited in order to establish the objectives of these events. In Belgium, for instance, members of the Flemish parliament requested that the “peace message: ‘Never again war’ [2] be central in the coordinated efforts to highlight the impact of the Great War. A report commissioned by the French president [3] considered the “teaching of disobedience with the causes, issues, and consequences of war” (p. 27) as one of the missions of the First World War centenary and as a cornerstone of education for peace. What these resolutions and recommendations have in common is the assumption that anti-war messages go a long way toward promoting peaceful attitudes, based on the notion that one can be either in favour of peace or in favour of war but not in favour of both at the same time. Such a conceptualization echoes the dominant perspective on the relationship between attitudes toward peace and war found in the psychological literature. In this perspective, peace and war attitudes are viewed as polar opposites along the same dimension [4,5] (for a brief review, see [6]).

According to Strachan [1], however, we cannot truly honour the fallen soldiers of WWI if we fail to acknowledge that certain values are worth fighting for and that the Great War was also waged for the cause of peace and freedom from tyranny. His analysis suggests that one can hold favourable attitudes toward both peace and war especially when war is seen as the best or only way to achieve one’s idealization of peace. Further supporting the possibility of a mutually non-exclusive relationship between attitudes toward peace and war, comparisons between cognitive/social representations of peace and war suggested that they are not complementary, with the former pertaining more to aspects within, and relations between, individuals, whereas the latter are typically applied to relations between nation-states [7] (for a brief review, see [8]). Hence, when peace and war are not merely represented as the absence of, respectively, war and peace, relatively independent attitudes toward these two objects might develop.

In order to test this possibility, Bizumic et al. [6] developed and validated the Attitudes toward Peace and War Scale (APWS), which consists of an equal number of items (eight, with four positively worded and four negatively worded) measuring each set of attitudes, giving a balanced measure of 16 items. They posited that attitudes toward peace and war are outcomes of different ideologies, values, and personality characteristics. Specifically, they argued that attitudes toward peace should be primarily and distinctly related to the abolition of structural kinds of violence [9], whereas attitudes toward war should be primarily and distinctly related to the acceptance of direct kinds of violence. Administering the scale to American and Danish respondents, they were able to substantiate these claims and found that, although negatively related, attitudes toward peace and war formed two distinct dimensions with unique antecedents and consequences. Their analyses indeed showed that attitudes toward peace were primarily associated with egalitarian ideological beliefs, the values of international harmony and equality, empathic concern for others, as well as a tendency to engage in pro-peace behaviours, whereas attitudes toward war were primarily associated with authoritarian ideological beliefs, the values of national strength and order, less personal distress, and a tendency to engage in pro-war behaviours. Their analyses further showed that, contrary to liberals, conservatives were more likely to favour both peace and war. Such a finding would not have been possible had they used traditional measures of attitudes toward peace and war, which either neglect attitudes toward peace or systematically pit peace against war [5,10].

Subsequent research has supported the convergent and divergent validity of the APWS by examining moral psychology [11], social representations [12], and personality variables [13]. For instance, using a measure of the Big Five Inventory, one of the most frequently used paradigms for personality traits, Blumberg, Zeligman, Appel, and Tibon-Czopp [13] found that the dimension of agreeableness, which expresses a prosocial and communal orientation toward others vs. antagonism, was associated (positively and negatively, respectively) with attitudes toward peace and attitudes toward war. In contrast, the dimension of openness to experience, which expresses the breadth, depth, and complexity of an individuals’ mental and experiential life, was (negatively) associated with attitudes toward war but not with attitudes toward peace.

Clearly, there is much to be learned from engaging with the complexity of generalized attitudes that people hold toward peace and war and by distinguishing between attitudes toward peace and war. The APWS is, to our knowledge, the only measure available for doing so, but it has yet to be validated in languages other than English. In this article, we aim to validate a French version of the APWS. The availability of this measure might foster the study of the relationship between attitudes toward peace and war in French-speaking samples and will allow for cross-cultural comparisons. To this end, the following steps were taken. In the first step, the original items of the scale were translated into French using a back-translation approach. In the second step, exploratory and confirmatory analyses were conducted in order to validate the French version and to assess the dimensionality of attitudes toward peace and war. In the third step, other psychometric properties of the French version were examined, such as the internal consistency, reliability, test-retest correlations, and method-effects. We specifically examined the role of socially desirable responding (SDR), as SDR has been identified as a potential confound in self-report research [14]. In the fourth and final step, we examined the criterion-related and known-groups kinds of validity of the French version of the APWS.

To assess the criterion-related validity, we first examined whether the scale related to political orientation. The most popular ideological dimensions proposed by theorists to capture the apparent diversity of political orientations and programs are the liberal-conservative and left-right dimensions [15]. A review of the literature [16] concluded that political conservatism consists of resisting change and justifying inequality, and is motivated by fear of threat, need for closure, and a lack of integrative complexity. Empirical research has furthermore shown that, in Western Europe, the left-right dimension has similar motivational underpinnings [17,18]. Conservatives and right-wingers could thus be expected to be less in favour of peace because they oppose equality, and more in favour of war because war provides for a “simpler” solution in threatening situations. Consistent with this assumption, militarism has been found to correlate positively [19], whereas a sense of peace responsibility has been found to correlate negatively, with conservatism [20]. We thus expected attitudes toward peace to relate negatively (hypothesis 1a) and attitudes toward war to relate positively (hypothesis 1b) to a right-wing political orientation.

Alongside political orientation, we sought to assess the criterion-related validity of the APWS with variables representing unique antecedents or consequences of attitudes toward peace and war. Concerning attitudes toward peace, we chose social dominance orientation (SDO) [21], empathic concern for others (EC) [22], and pro-peace behavioural intentions because these variables were expected to relate primarily to attitudes toward peace. Bizumic et al. [6] have argued that attitudes toward peace are primarily and distinctly related to the abolition of structural kinds of violence. Accordingly, individuals who approve of hierarchy and inequality between social groups should be less supportive of peace than those who endorse egalitarian beliefs. A widely studied ideological belief that approves of intergroup inequalities and dominance is social dominance orientation (SDO) [21]. Specifically, we expected attitudes toward peace to relate negatively to SDO (hypothesis 2). We also expected attitudes toward peace to relate positively to empathy [22], especially its EC component (hypothesis 3a), as EC is most directly concerned with sympathy for the suffering of the unfortunate [23]. Finally, we expected attitudes toward peace to be positively associated with a willingness to promote peace (hypothesis 4a).

Concerning attitudes toward war, and in line with previous work [24], we examined the association between attitudes toward war and national identification. Specifically, we expected attitudes toward war to be positively associated with national identification (hypothesis 5) because an enhanced view of one’s country could contribute to a greater propensity toward outgroup conflict, especially in threatening situations [25]. Next, despite empathy having been associated with attitudes toward both peace and war, we expected its personal distress (PD) [23] component to be primarily and negatively associated with attitudes toward war (hypothesis 3b), given the extreme and dramatic harm that results from wars, and strong anxiety in threatening circumstances associated with this aspect of empathy. We also expected attitudes toward war to relate positively to a willingness to promote war (hypothesis 4b). Finally, Bizumic et al. [6] have argued that attitudes toward war are primarily and distinctly related to the acceptance of direct kinds of violence. Accordingly, individuals who uncritically defend their ingroup and its interests or traditions, and who show a preference for an aggressive way of dealing with out-group threats should be more supportive of war as an instrument to alleviate threats. A widely studied ideological belief that expresses the motivational goal of ingroup security and order generated by a view of the social world as a dangerous and threatening place is right-wing authoritarianism (RWA) [26]. Specifically, we expected attitudes toward war to relate positively to RWA (hypothesis 6).

In terms of known-groups validity, we examined the associations of the APWS with gender. One of the first differences observed between men and women in socio-political attitudes concerns the tendency of women to be more accepting of compromise and less supportive of violence and war than men [27]. The replication of this gender gap across a wide range of studies [28] has led researchers to postulate a unique relationship between women and peace, which has been called the women and peace hypothesis [29]. Consequently, we expected women to be more and less favourable, respectively, toward peace and war than men (hypotheses 7a and 7b).

## Method

### Procedure and participants

Five samples (total N = 808) were used throughout the four steps of this research: four student samples and one nonstudent sample. Sample 1 was collected between March and April 2014 as part of a larger cross-cultural study (for further details on the sampling and methodology, see [30]) in which university students participated by completing an online questionnaire in exchange for course credits. For the purposes of the present research, we analysed the answers of 302 participants from French-speaking Belgium and France, irrespective of their nationality. Sample 2 included 181 participants recruited between March and April 2015 via a Facebook page joined by students (currently enrolled or former) of the authors’ university, 107 of which were first year students who participated in exchange for course credits, whereas the remaining 74 were former students who participated on a voluntary basis. Fourteen participants who failed to answer correctly one or more of the three instructed response items [31] that were incorporated in the online questionnaire were excluded from the analyses. Sample 3 included 83 students from a French-speaking Belgian secondary school who were invited to complete a paper-and-pencil questionnaire on 3 occasions between February and March 2015: 15 days before the visit of an exhibition (about WWI), at the end of the visit, and 15 days after the visit. This sample was collected to appraise the test-retest reliability of the scale. To this end, we analysed the answers provided by the participants on the two first occasions. Two students participated only on the first occasion, as a consequence of which they were not included in the calculation of the test-retest correlations. The dataset was further corrected for missing values (<1%) using expectation maximization. Sample 4 comprised 236 nonstudent adults. They were recruited between March and April 2016 via an email sent to the parents of a French-speaking Belgian scouts unit. Data were collected via a voluntary and confidential online questionnaire. Of the 255 persons who received the email, 236 (93%) started the questionnaire, and 195 completed it. Little’s test [32] for missing completely at random (MCAR) was nonsignificant, *χ*^*2*^(19) = 6.92, *p* = .99, indicating that the rate of missing values on one variable was unrelated to the other measured or unmeasured variables [33]. Consequently, data from non-completers were dropped [34]. Nine participants who failed to answer correctly one or more of the two instructed response items that were incorporated in the questionnaire were additionally excluded from the analyses. Sample 5 included 77 first-year psychology students, recruited between May and June 2017, who participated in exchange for course credits. Two participants who did not provide demographic information and 3 participants who failed to answer correctly one or more of the five instructed response items that were incorporated in the online questionnaire were excluded from the analyses. In all samples except sample 3, participants were presented with an online informed consent form and were requested to give their consent by clicking on a radio button before accessing the questionnaire. In sample 3, written informed consent was obtained from participants before the questionnaire was handed out to them. Information about participants’ demographics is displayed in Table 1. Table 2 provides an overview of how the samples were used throughout the four steps, along with the variables assessed in each sample.

**Table 1:** Demographic characteristics of the participants in the five samples.

	Sample 1	Sample 2	Sample 3	Sample 4	Sample 5
*N*	302	167	81	186	72
Gender					
Male (%)	27	32	43	25	28
Female (%)	73	67	47	75	72
Other (%)	0	1	10	0	0
Age					
Mean (years)	21.07	21.30	16.43	47	21.33
SD (years)	5.39	3.70	.74	10.87	4.79
Range (years)	18–62	16–46	15–19	21–74	18–50
Nationality					
Belgian (%)	57	74	89	91	81
French (%)	34	11	1	5	7
Other (%)	9	15	10	4	12
Education					
Primary or secondary (%)	69	74	100	20	100
Vocational (%)	0	5	—	28	—
University (%)	31	21	—	52	—
Occupation					
Student (%)	100	87	100	—	100
Professional (%)	—	10	—	81	—
Manager (%)	—	0	—	11	—
Other^a^ (%)	—	3	—	8	—

*Note*.

^a^ Includes unemployed, blue collar workers, and administrative personnel.

**Table 2:** Overview of the samples throughout the four steps.

	Sample 1	Sample 2	Sample 3	Sample 4	Sample 5
Step I					
Scale backtranslation					
Step II					
Factor structure	X	X		X	
Step III					
Intercorrelations	X	X	X	X	X
Internal consistency	X	X	X	X	X
Test-retest			X		
Method effects				X	
Step IV					
Criterion-related validity					
Political orientation	X	X		X	X
National identification (i.e., attachment and glorification)	X				
Social dominance orientation		X			X
Empathy (i.e., personal distress and empathic concern)		X			X
Behavioral intentions					
Willingness to fight for one’s country	X				
Pro-peace and pro-war behavioral intentions		X			X
Right-wing authoritarianism					X
Known-groups validity					
Gender	X	X	X	X	X

### Measures

Unless otherwise indicated, a response scale ranging from 1 (= Strongly disagree) to 7 (= Strongly agree) was used for all measures.

#### Political orientation

Political orientation was assessed in all samples except sample 3 with a single-item measure: Participants were asked to place themselves on a scale ranging from 1 (= Far left) to 7 (= Far right). This measure is highly similar to the traditional single-item measure of liberal vs. conservative political orientation used in much previous research [15]. Research with French-speaking samples in Belgium and Switzerland has supported the validity of this singe-item measure by showing that it correlates with various socio-political issues [35,36]. *M*_*sample1*_ = 3.52 (*SD* = 1.45), *M*_*sample2*_ = 3.32 (*SD* = 1.26), *M*_*sample4*_ = 3.17 (*SD* = 1.17), and *M*_*sample5*_ = 3.43 (*SD* = 1.35).

#### National identification

National identification was assessed in sample 1 with a scale developed in Israel by Roccas, Klar, and Liviatan [37], which distinguishes between attachment (8 items, e.g., ‘It is important to me to contribute to my nation’; *M* = 4.29, *SD* = 1.25, *α* = .90) and glorification (7 items, e.g., ‘Other nations can learn a lot from us’; *M* = 3.20, *SD* = .86, *α* = .74). Items were translated into French and adapted to refer to Belgium or France. One item from the original glorification sub-scale (i.e., ‘The IDF is the best army in the world’) was not retained because it was judged irrelevant in the Belgian and, to a lesser extent, in the French contexts.

#### Social dominance orientation

SDO was assessed in samples 2 and 5 with 10 items (e.g., ‘No one group should dominate in society’; reversed, sample 2: *M* = 2.29, *SD* = .94, *α* = .82; sample 5: *M* = 2.15, *SD* = .97, *α* = .85) from Sidanius and Pratto [38] translated and validated in French [39].

#### Empathy

The PD and EC components of the Interpersonal Reactivity Index [22] were assessed in samples 2 and 5 with a validated French version [40]. Both the PD (e.g., ‘In emergency situations, I feel apprehensive and ill at ease’; sample 2: *M* = 3.40, *SD* = 1.01, *α* = .79; sample 5: *M* = 3.58, *SD* = 1.07, *α* = .78) and EC (e.g., ‘I often have tender, concerned feelings for people less fortunate than me’; sample 2: *M* = 5.26, *SD* = .97, *α* = .80; sample 5: *M* = 5.31, *SD* = .98, *α* = .77) components consisted of 7 items. Participants indicated their level of agreement with each item on a scale ranging from 1 (= Does not describe me well) to 7 (= Describes me very well).

#### Behavioral intentions

Behavioral intentions were assessed in two ways. In sample 1, we used a single-item appraising participants’ willingness to fight for their country: ‘Of course, we all hope that there will not be another war, but if it were to come to that, would you be willing to fight for your country?’ and provided participants with a response scale ranging from 1 (= Not at all) to 7 (= Totally; *M* = 2.97, *SD* = 1.89). Previous research has shown that this item correlates with social psychological variables, including those among French respondents [41].

In samples 2 and 5, participants were presented with items measuring behavioral intentions that were pro-peace (5 items, e.g., ‘I would join a human barricade to promote peace’; sample 2: *M* = 4.41, *SD* = 1.19, *α* = .71; sample 5: *M* = 4.61, *SD* = 1.34, *α* = .81) and pro-war (5 items, e.g., ‘If it is in my country’s best interest, I would write letters to officials to support my country going to war’; sample 2: *M* = 2.37, *SD* = 1.17, *α* = .77; sample 5: *M* = 2.49, *SD* = 1.06, *α* = .67), which were taken from Bizumic et al. [6]. Items were translated to French.

#### Right-wing authoritarianism

RWA was assessed in sample 5 with 20 items (e.g., ‘Our country needs a powerful leader, in order to destroy the radical and immoral currents prevailing in society today’; *M* = 2.68, *SD* = .81, *α* = .84) from Altemeyer [26] translated and validated in French [42].

#### Socially desirable responding

SDR was assessed in sample 4 with the lie scale, which is part of the abbreviated form of the revised Eysenck Personality Questionnaire (EPQ-A) [43], as translated and validated in French [44]. The lie scale has already been used successfully to measure the extent to which SDR biased participants’ answers to questions about their socio-political attitudes [45]. In its abbreviated form, it consists of 6 items with a ‘Yes/No’ response scale, and is written in such a way that a given response is socially desirable but highly unlikely, as in the item ‘Do you always practice what you preach?’. Socially desirable answers were coded 1 and the socially undesirable answers 0. Answers were then summed to obtain a scale ranging from 0 to 6 (*M* = 3.38, *SD* = 1.71).

## Results

### Step I: Translation

The APWS was translated according to guidelines suggested by Brislin [46], beginning with a parallel back-translation procedure: In each of two pairs of bilingual psychologists (including the last author) working independently, one translator was asked to translate the original item into French and the other to translate it back into English. Out of the 16 translated items, four were not equivalent across pairs of translators. Based on feedback from the translators, we improved the instructions and carried out an iterative back-translation procedure [46]. New pairs of bilingual psychologists were solicited with the second pair working with the back-translated items of the first pair. Next, an English professor and academic head of the languages department of a Belgian (French-speaking) university rated the extent to which each of the final back-translated items was a very bad, bad, good, very good or excellent cultural (as opposed to literal) equivalent of the original item. Although one item (i.e., item 8 of the war subscale; see S1 File) received a rating below very good, we kept it because the English professor considered the two translated items to be very good translations of the original item. The exact wording of the translated items in the studies as well as of the original and back-translated items are set out in the S1 File. Concerning the response scale, whereas the original APWS was assessed on a 9-point scale, we used a 7-point scale ranging from 1 (= Strongly disagree) to 7 (= Strongly agree).

### Step II: Factor structure

To examine the factor structure of the scale, we first conducted an EFA (principal axis factoring) in all samples except samples 3 and 5 because, unlike the three other samples, they did not meet MacCallum, Widaman, Zhang, and Hong’s [47] minimum requirements for factor analysis. These requirements state that samples should be in the range of 100 to 200 when most communalities are in the range of .50 and factors are well-determined, all conditions that were met in [6]. We used an oblique rotation (i.e., direct oblimin; as in [6]) with Kaiser normalisation and selected the optimal number of factors to be retained based on the scree plot and the Schwarz Bayesian information criterion (BIC) as cited in [48]. Across samples, the scree plot and the BIC consistently supported a two-factor solution.

The factor structure of the APWS items was further explored via CFA using maximum-likelihood estimation with Stata 14 [49]. Model fit was evaluated with the following goodness of fit indices: the standardized root mean square residuals (SRMR), the root mean square error of approximation (RMSEA), and the comparative fit index (CFI). SRMR and a RMSEA below .10 together with a CFI above .90 indicate good fit, whereas SRMR below .8, a RMSEA not superior to .05, and a CFI above .95 indicate excellent fit [50]. *χ*^*2*^ difference tests were used to identify the best fitting model [34]. As a preliminary analysis revealed non-normality at the univariate and multivariate levels, the Satorra-Bentler scaling method, as cited in [51], was used. In each sample, a one-factor model where items measuring attitudes toward peace and war load on a single latent variable (Model 1) was compared to an unconstrained two-factor model (i.e., the covariances between the latent variables are freely estimated) where items measuring attitudes toward peace and items measuring attitudes toward war load on two distinct latent variables (Model 2).

Results in the first sample revealed that the fit was substantially better for Model 2, which had two fit indices out of three that were good (*χ*^*2*^_*SB*_(103) = 221.98, *p* < .001, SRMR = .07, RMSEA_*SB*_ = .06, *p* < .01, CFI_*SB*_ = .89, Δ *χ*^*2*^_*SB*_(1) = 258.00, *p* < .001), than for Model 1, which had unacceptable fit indices (*χ*^*2*^_*SB*_(104) = 479.98, *p* < .001, SRMR = .10, RMSEA_*SB*_ = .11, *p* < .001, CFI_*SB*_ = .65). However, modification indices suggested adding covariance between the error terms of 4 pairs of items (between items 1 and 3 and between items 2 and 3 of the peace subscale, on the one hand, and between items 4 and 5 and between items 6 and 7 of the war subscale, on the other hand). Because the items composing each of these pairs loaded on the same latent variable, adding their covariance to Model 2 was not considered problematic. This revised model had excellent fit indices and fitted the data substantially better (*χ*^*2*^_*SB*_(99) = 157.03, *p* < .01, SRMR = .06, RMSEA_*SB*_ = .04, *p* = .382, CFI_*SB*_ = .95, Δ *χ*^*2*^_*SB*_(4) = 64.95, *p* < .001) than Model 2. Within this revised model, all items had significant loadings (ranging from .25 to .78, *p* < .001, with an average loading of .55; see S3 File for further details on CFA loadings).

Since the areas of local misfit may simply be a reflection of the idiosyncratic characteristics of a particular data set, it was important to make sure they were consistent across samples [52]. In other words, the areas of misfit were first diagnosed with the first sample and were then cross-validated using samples 2 and 4. In both these latter samples, the revised Model 2 had all good fit indices and fitted the data substantially better (sample 2: *χ*^*2*^_*SB*_(99) = 139.82, *p* < .01, SRMR = .07, RMSEA_*SB*_ = .05, *p* = .096, CFI_*SB*_ = .92, Δ *χ*^*2*^_*SB*_(4) = 17.84, *p* < .01; sample 4: *χ*^*2*^_*SB*_(99) = 138.42, *p* < .01, SRMR = .07, RMSEA_*SB*_ = .05, *p* = .214, CFI_*SB*_ = .92, Δ *χ*^*2*^_*SB*_(4) = 11.78, *p* < .05) than Model 2 (sample 2: *χ*^*2*^_*SB*_(103) = 157.66, *p* < .001, SRMR = .08, RMSEA_*SB*_ = .06, *p* < .05, CFI_*SB*_ = .89; sample 4: *χ*^*2*^_*SB*_(103) = 150.20, *p* < .01, SRMR = .07, RMSEA_*SB*_ = .05, *p* = .136, CFI_*SB*_ = .91), which itself fitted the data substantially better (sample 2: Δ *χ*^*2*^_*SB*_(1) = 126.82, *p* < .001; sample 4: Δ *χ*^*2*^_*SB*_(1) = 85.56, *p* < .001) than Model 1 (sample 2: *χ*^*2*^_*SB*_(104) = 284.48, *p* < .001, SRMR = .10, RMSEA_*SB*_ = .10, *p* < .001, CFI_*SB*_ = .65; sample 4: *χ*^*2*^_*SB*_(104) = 235.76, *p* < .001, SRMR = .09, RMSEA_*SB*_ = .08, *p* < .001, CFI_*SB*_ = .75).

It should however be noted that exploratory analyses (e.g., EFA) indicated that one item (i.e., item 8 of the war subscale) consistently failed to meet the cut-off criterion of .30 set for corrected item-total correlations, which is indicative of insufficient variance [53]; that four items (items 6 and 8 of the peace subscale and items 6 and 7 of the war subscale) had consistent weak pattern coefficients (i.e., < .40; [50]) on their intended factor; and that one item (item 2 of the peace subscale) had weak pattern coefficients on its intended factor in two samples and had even a larger cross-loading in one sample (see S2 File for further details on EFA loadings). The factor structure of a shortened form of the scale excluding the above items was examined via EFA. Across samples, the scree plot and the BIC consistently supported a two-factor solution with peace and war attitudes items loading moderately (i.e., > .4) to strongly on their intended factor.

The factor structure of this shortened APWS was further explored via CFA. We specifically compared a revised Model 2, in which we again allowed the error terms of items 1 and 3 of the peace subscale and the errors terms of items 4 and 5 of the war subscale to covary, to Model 1. Results revealed once again that the fit was substantially better for the revised Model 2, which had excellent fit indices (sample 1: *χ*^*2*^_*SB*_(32) = 57.45, *p* < .01, SRMR = .05, RMSEA_*SB*_ = .05, *p* = .193, CFI_*SB*_ = .97, Δ *χ*^*2*^_*SB*_(3) = 278.91, *p* < .001; sample 2: *χ*^*2*^_*SB*_(32) = 50.17, *p* < .05, SRMR = .06, RMSEA_*SB*_ = .06, *p* = .104, CFI_*SB*_ = .95, Δ *χ*^*2*^_*SB*_(3) = 124.07, *p* < .001; sample 4: *χ*^*2*^_*SB*_(32) = 39.05, *p* = .187, SRMR = .05, RMSEA_*SB*_ = .03, *p* = .494, CFI_*SB*_ = .98, Δ *χ*^*2*^(3) = 101.42, *p* < .001), than for Model 1, which had all unacceptable fit indices (sample 1: *χ*^*2*^_*SB*_(35) = 336.36, *p* < .001, SRMR = .13, RMSEA_*SB*_ = .17, *p* < .001, CFI_*SB*_ = .62; sample 2: *χ*^*2*^_*SB*_(35) = 174.87, *p* < .001, SRMR = .13, RMSEA_*SB*_ = .16, *p* < .001, CFI_*SB*_ = .63; sample 4: *χ*^*2*^_*SB*_(35) = 140.47, *p* < .001, SRMR = .11, RMSEA_*SB*_ = .13, *p* < .001, CFI_*SB*_ = .72). All items had significant loadings (ranging from .33 to .85, *p* < .001 with an average loading of .63 in all 3 analyzed samples). Despite its superior discriminant validity, the shortened form yielded a pattern of results highly similar to the long form in terms of reliability (see below) and both criterion-related (see S4 File) and known-groups (see S5 File) validity.

### Step III: Intercorrelations, reliability, and method effects

Across the three samples used for the CFA, the latent variables of attitudes toward peace and attitudes toward war correlated on average -.50. The internal consistency of the peace and war subscales in the five samples can be considered acceptable (i.e., .74 in both cases). Test-retest correlations were calculated in sample 3 and were .65 and .75 for the peace and war subscales, respectively. Concerning the shortened form of the APWS, the latent variables of attitudes toward peace and attitudes toward war correlated on average -.42; the internal consistency of the peace and war subscales in the five samples were, on average, .74 and .76, respectively; and test-retest correlations were .68 and .68 for the peace and war subscales, respectively.

Although the scale was reliable, method effects may have contaminated our participants’ answers. To examine this issue, we investigated the effect of SDR on our participants’ answers in sample 4. As can be seen in Table 3, neither attitudes toward peace nor attitudes toward war were associated with SDR. Moreover, the associations between the APWS and political orientation (see next section) remained unchanged even after controlling for SDR (we report the results for the shortened form in S4 File).

**Table 3:** Semi-partial correlations between measures in samples 1, 2, 4, and 5.

	Peace subscale				War subscale			
	Sample 1	Sample 2	Sample 4	Sample 5	Sample 1	Sample 2	Sample 4	Sample 5
Political orientation	-.21**	-.23**	-.18*	-.21	.15*	.12	.14	.16
Attachment	.11				.21***			
Glorification	.12*				.19**			
SDO		-.41***		-.24*		.25***		.35***
PD		.12		.13		-.02		.08
EC		.33***		.33**		-.12		-.03
Willingness to fight for one’s country	.00				.32***			
PBI		.56***		.44***		-.05		-.13
WBI		.03		.13		.35***		.28*
RWA				.16				.42***
SDR			.03				.06	

*Note*. SDO = social dominance orientation. PD = personal distress. EC = empathic concern. PBI = pro-peace behavioral intentions. WBI = pro-war behavioral intentions. RWA = right-wing authoritarianism. SDR = socially desirable responding.

* *p* < .05.

** *p* < .01.

*** *p* < .001.

### Step IV: Criterion-related and known-groups validity

#### Criterion-related validity

Having established the two-factor structure and the reliability of the French version of the APWS, we proceeded to assess its criterion-related validity. Rather than reporting zero-order correlations, which might sometimes obscure relationships when there are strongly related measures, we decided to regress the criterion variables on attitudes toward peace and war in a series of simultaneous regression analyses. This allowed us to test the relationship between attitudes toward peace and a given criterion after the overlap between attitudes toward peace and war had been taken into account, and vice and versa (for a similar approach, see [54]). We then compared the semipartial correlations between attitudes toward peace and a given criterion variable and attitudes toward war and the same criterion variable using the procedure of Meng, Rosenthal, and Rubin [55] as implemented in the MALG.FOR program [56]. Before proceeding to the comparison of correlations that were in opposite directions, we reverse coded the variable that was unique to the negative correlation. Except for political orientation and socially desirable responding, we used one-tailed tests given our a priori predictions concerning which criterion variables should be related to attitudes toward peace and war.

The semipartial correlations between the APWS and the criterion-related variables are presented in Table 3. As can be seen, attitudes toward peace and attitudes toward war were correlated, as expected (hypotheses 1a and 1b), negatively and positively, respectively, with political orientation. Although the semipartial correlations between attitudes toward war and political orientation failed to reach statistical significance in three samples out of four, comparisons of the semipartial correlations revealed that political orientation was not more strongly associated with attitudes toward peace than with attitudes toward war (.49^ns^ ≤ all *z*s ≤ 1.30^ns^).

In line with hypothesis 5, attitudes toward war correlated positively with national identification (i.e., attachment and glorification) but attitudes toward peace also correlated positively with glorification. Attachment was more strongly associated with attitudes toward war than with attitudes toward peace (*z* = 1.66, *p* < .05) but glorification was not (*z* = 1.16^ns^). As expected, attitudes toward peace correlated negatively with SDO, supporting hypothesis 2. Surprisingly, attitudes toward war correlated positively with SDO. Moreover, SDO was not more strongly associated with attitudes toward peace than with attitudes toward war (*z*_*sample2*_ = 1.40^ns^; *z*_*sample5*_ = -1.11^ns^). Also unexpected, attitudes toward war did not correlate with PD. Hypothesis 3b was thus not supported.

As expected, attitudes toward peace correlated positively with EC, supporting hypothesis 3a. Besides, they were more strongly associated with EC than attitudes toward war in both samples 2 and 5 (*z*_*sample2*_ = 2.52, *p* < .01; *z*_*sample5*_ = 2.91, *p* < .01). In line with hypotheses 4a and 4b, attitudes toward peace correlated positively with pro-peace behavioral intentions, whereas attitudes toward war correlated positively with willingness to fight for one’s country and pro-war behavioral intentions. Moreover, attitudes toward war were more strongly associated with willingness to fight for one’s country (*z* = 5.32, *p* < .001) and pro-war behavioral intentions (*z*_*sample2*_ = 3.81, *p* < .001; *z*_*sample5*_ = 1.47^ns^) than were attitudes toward peace. Similarly, attitudes toward peace were more strongly associated with pro-peace behavioral intentions than were attitudes toward war (*z*_*sample2*_ = 6.39, *p* < .001; *z*_*sample5*_ = 3.11, *p* < .001). Finally and in line with hypothesis 6, attitudes toward war correlated positively with RWA and were more strongly associated with RWA than attitudes toward peace (*z* = 2.61, *p* < .01).

#### Known-groups validity

The mean differences and effect sizes for gender are presented in Table 4 (for the shortened form, see S5 File). As can be seen, hypotheses 7a and 7b were partially supported as women had, respectively, more and less favourable attitudes toward peace and war than men in four out of the five samples. Single-paper meta-analyses [57] estimated the effect of gender on attitudes toward peace at .33 (*SE* = .091; *z* = 3.58, *p* < .001) and on attitudes toward war at .48 (*SE* = .101; *z* = 4.82, *p* < .001). For the shortened form, the effect of gender on attitudes toward peace across the five samples was estimated at .34 (*SE* = .103; *z* = 3.32, *p* < .001) and on attitudes toward war at .65 (*SE* = .129; *z* = 5.02, *p* < .001).

**Table 4:** Mean differences and effects sizes for gender in samples 1 to 5.

Gender	Men	Women
APWS and samples	*η*^*2*^	*η*^*2*^
Peace subscale		
Sample 1	5.38 (1.05)	5.65 (0.84)
	.02*	
Sample 2	4.98 (1.11)	5.41 (0.87)
	.04**	
Sample 3	5.30 (0.93)	5.70 (0.67)
	.06*	
Sample 4	5.73 (0.94)	5.75 (0.97)
	.00	
Sample 5	4.92 (0.61)	5.56 (0.86)
	.11***	
War subscale		
Sample 1	3.27 (1.13)	2.69 (0.96)
	.06***	
Sample 2	3.12 (1.00)	2.57 (0.98)
	.06**	
Sample 3	3.11 (0.96)	2.43 (0.77)
	.14**	
Sample 4	2.62 (1.09)	2.61 (1.10)
	.00	
Sample 5	3.42 (0.92)	2.75 (1.01)
	.08***	

*Note*. Standard deviations are between brackets. Effect size was calculated using *η*^*2*^ = eta squared.

* *p* < .05.

** *p* < .01.

*** *p* < .001.

## Discussion

The idea that attitudes toward peace and war are two polar opposites along the same continuum has pervaded many of the measures used in the empirical literature [4,5]. Running counter this trend, Bizumic et al. [6] developed and validated the APWS and found that, although negatively related, attitudes toward peace and war formed two distinct dimensions with different antecedents and consequences. In this article, we reported the results of research aimed at validating a French version of this scale. Results across five samples (total N = 808) provided good support for the psychometric properties of the French version of the APWS. Consistent with the nomological network within which the scale is positioned, attitudes toward peace and war were found to represent negatively related, yet distinct, constructs. Furthermore, the subscales proved to have good reliability and participants’ answers to the items were not significantly affected by socially desirable responding.

In addition and providing criterion-related evidence for the French version of the APWS, we found that attitudes toward peace were related to political orientation, SDO, empathic concern, and pro-peace behavioural intentions in a predictable way [20]. Specifically, as expected, attitudes towards peace were negatively correlated with (a right-wing) political orientation and SDO, and positively correlated with empathic concern and pro-peace behavioral intentions. In a similar vein, we found that attitudes toward war were related to political orientation, national identification, pro-war behavioral intentions, and RWA in a consistent way [24]. Specifically, as expected, attitudes toward war were positively related to (a right-wing) political orientation, national identification, pro-war behavioral intentions, and RWA.

Unexpectedly, however, attitudes toward war were not related to personal distress and were positively related to SDO. A possible explanation for the former finding could lie in the fact that a proneness to feel anxious in threatening situations may negatively affect attitudes towards war only when individuals are thinking of the extreme and dramatic harm that results from wars. Previous research suggests that this may not always be the case. Indeed, scholars have classified individuals’ representations of war into three broad categories of meaning: a) armed conflict, b) direct violence, and c) indirect violence [12]. The first category is more descriptive and includes objects, activities, and actors of war. Yet it is prevalent in individuals’ representations of war [58]. A possible explanation for the finding that attitudes toward war are positively related to SDO also stresses the value of studying attitudes toward war in conjunction with representations of war. Pratto et al. [21] have shown that people high in SDO tend to approve of different kinds of hierarchy-enhancing attitudes, including warlike and militaristic attitudes, but only when these can enhance or preserve group-based hierarchies. We could thus expect an interaction effect such that SDO will be (positively) related to attitudes toward war when indirect violence (which includes social injustice and global economic oppression [12]) is the most dominant or salient category of meaning in individuals’ representations of war.

More recently, Ho et al. [54] substantiated the claim that SDO consists of two complementary dimensions: SDO-Dominance (i.e., a preference for a society where some groups forcefully dominate others) and SDO-Egalitarianism (i.e., a preference for nonegalitarian intergroup relations through subtle hierarchy-enhancing social policies). Using SDO as a unitary construct in our research might thus have obscured the possibility that attitudes toward peace and war are more related to, respectively, SDO-Egalitarianism and SDO-Dominance. The scales developed by Ho et al. to disentangle these two dimensions have not been validated in French yet, preventing us from testing these possibilities. We would like to encourage further research to examine these issues in greater detail.

Consistent with the women and peace hypothesis [28] and providing known-groups validity to the French version of the APWS, we found that women had, respectively, more and less favourable attitudes toward peace and war than men. Yet, the gender gap failed to replicate in one sample. This result could, of course, be a false negative [59] but other explanations are possible. According to social role theory [60], gender gaps in socio-political attitudes occur because men and women do not occupy the same social (i.e., professional, private, and gender) roles. Because women more often occupy social roles promoting communal values (e.g., housewives), they tend to develop attitudes that are, respectively, more and less compatible with support for compromise and violence than men, who more often occupy social roles promoting instrumental values (e.g., managers). The sample in which the gender gap did not replicate was composed almost exclusively of working adults. Moreover, female and male participants were equally divided into different professional roles and sectors, and all were parents. It is thus possible that female and male participants had similar attitudes toward peace and war because they occupied similar professional and private roles. The APWS may assist future research aimed at testing this possibility.

As a cautionary note, although the hypothesized two-factor model had acceptable fit indices in the three samples in which we conducted factor analyses, six items did not perform well (i.e., they had insufficient variance or pattern coefficients < .40). One might invoke cultural or contextual differences to explain this result. For instance, item 2 of the peace subscale is presumably located far on the negative end of the attitudes toward peace continuum. In other words, agreeing with this item might reveal a more negative attitude than agreeing with item 7. In countries where the political culture is conservative and tends to justify violence, such as in the United States [61], and in countries that are participating in a war effort, such as Denmark at the time when Bizumic et al. [6] collected their data, we might expect item 2 to perform better. Indeed, in these contexts the championing of peace is usually less socially desirable, which could translate into higher levels of agreement with this item and increased variability in responses. In Belgium, a country that was ranked as the 18th most peaceful country on the 2016 Global Peace Index (compared to the United States which was ranked 103rd on the same index) [62], and which was not involved in a war effort at the time we collected our data [63], this item may have seemed culturally and/or contextually irrelevant. It is also possible that the syntax of some items, such as item 8 of the war subscale, was too complex once translated into French. Another explanation might be that the change we have made to the response scale, that is, a 7-point scale instead of a 9-point scale, reduced variability in the responses, which may have proved problematic for the above items. More studies are needed to investigate this issue further.

Representing another important contribution of the present research, we demonstrated that a shortened form of the French version of the APWS had superior discriminant validity but otherwise similar psychometric properties to the long form, thereby providing researchers with a measure to use in cases where space/time constraints are a pressing issue. However, it is worth emphasizing that the results of the shortened form only apply to the French version of the scale and not to the English version.

One limitation of the present research pertains to the fact that the criterion-related validity of the APWS was established by means of correlations with variables found in past research to represent antecedents and consequences of attitudes toward peace and war. However, the cross-sectional nature of our data does not allow us to draw inferences about the direction of effects, which only longitudinal or experimental data could.

Finally, future studies might also seek to assess the predictive validity of the APWS by examining the relative contribution of attitudes toward peace and war in the prediction of various outcomes such as attitudes toward specific wars, policy preferences, and voting behavior. Alternatively, and in line with Carnagey and Anderson [64] showing that large-scale events that are relevant to issues of peace or war can change attitudes toward war, future research may explore whether certain events or interventions (i.e., commemorations) affect attitudes toward peace and war differently, such that each set of attitudes relates to unique features of individuals’ context [6]. The APWS allows for testing this assumption.

As concluding remarks, the present results support the psychometric properties of the French version of the APWS in five samples with a total of 808 participants varying in nationality, education, occupation, age, and gender. The availability of this measure may help stimulate the study of peace psychology in cross-cultural designs or intervention studies, and may contribute to a more comprehensive and nuanced answer to the question of whom, when, why, and under what circumstances are people willing to promote peace and/or war. Nevertheless, the results also underline the fact that the inclusion of some items could be questioned for the factorial structure of the measure, opening up the opportunity for improving the scale.

## Supporting information

S1 File APW scale: Original english items, French translations, and back-translations. (DOCX)

S2 File EFA pattern loadings based on oblimin rotation with Kaiser normalization of items measuring attitudes toward peace and war in samples 1, 2, and 4. (DOCX)

S3 File CFA standardized loadings of items measuring attitudes toward peace and war in samples 1, 2, and 4. (DOCX)

S4 File Semi-partial correlations between measures for the shortened form of the APWS in samples 1, 2, 4, and 5. (DOCX)

S5 File Mean differences and effects sizes for gender for the shortened form of the APWS in samples 1 to 5. (DOCX)
