# Supplementary material for: Are attitudes toward peace and war the two sides of the same coin? Evidence to the contrary from a French validation of the Attitudes Toward Peace and War Scale
Source: PLoS One. 2017 Sep 11;12(9):e0184001. doi: 10.1371/journal.pone.0184001 (PMC5593180; doi:10.1371/journal.pone.0184001)
Supplement: S1 File — (DOCX) [file pone.0184001.s001.docx]

| **S1 File.** | |
| --- | --- |
| APW Scale: Original English Items, French Translations, and Back-Translations. | |
| **Peace subscale** | |
| 1. Our country’s first priority should be world peace.^a^  French translation: La première priorité de notre pays devrait être la paix dans le monde.  Back-translation: The first priority of our country should be world peace. | |
| 2. People who place a high value on peace are usually weak and cowardly.  French translation: Les gens qui attachent une grande importance à la paix sont généralement faibles et lâches.  Back-translation: People who attach much importance to peace are generally weak and cowardly. | |
| 3. We must devote all our energy to securing peace throughout the world.^a^  French translation: Nous devons consacrer toute notre énergie à assurer la paix dans le monde entier.  Back-translation: We should invest all our energy to ensure peace throughout the world. | |
| 4. I believe that peace is extremely important.^a^  French translation: Je crois que la paix est extrêmement importante.  Back-translation: I believe that peace is extremely important. | |
| 5. Peace brings out the best qualities in a society.^a^  French translation: La paix fait ressortir les meilleures qualités d’une société.  Back-translation: Peace brings out the best qualities of a society. | |
| 6. There are many things in life that are more important than peace.  French translation: Il y a beaucoup de choses dans la vie qui sont plus importantes que la paix.  Back-translation: There are many things in life that are more important than peace. | |
| 7. In general, I am not too concerned about peace in the world.^a^  French translation: En général, je ne suis pas trop concerné-e par la paix dans le monde.  Back-translation: As a general rule, I am not very concerned about world peace. | |
| 8. The preservation of peace may sometimes hinder our country’s progress.  French translation: Le maintien de la paix peut parfois entraver les progrès de mon pays.  Back-translation: Maintaining peace can sometimes obstruct the progress of my country. | |
| **War subscale** | |
| 1. There is no conceivable justification for war.^a^  French translation: Il n’y a pas de justification concevable à la guerre.  Back-translation: There is no conceivable justification for war. | |
| 2. War is sometimes the best way to solve a conflict.^a^  French translation: La guerre est parfois la meilleure manière de résoudre un conflit.  Back-translation: Sometimes war is the best solution for resolving a conflict. | |
| 3. War is a futile struggle resulting in self-destruction.^a^  French translation: La guerre est une lutte futile résultant en l’autodestruction.  Back-translation: War is a futile struggle that results in self-destruction. | |
| 4. Under some conditions, war is necessary to maintain justice.^a^  French translation: Sous certaines conditions, la guerre est nécessaire pour maintenir la justice.  Back-translation: Under certain conditions, war is necessary for maintaining justice. | |
| 5. Although war is terrible, it has some value.^a^  French translation: Bien que la guerre soit terrible, elle a une certaine utilité.  Back-translation: Even if war is terrible, it has a certain use. | |
| 6. The evils of war are greater than any possible benefits.  French translation: Les côtés néfastes de la guerre sont plus grands que ses bénéfices éventuels.  Back-translation: The negative aspects of war are greater than the possible advantages. | |
| 7. War breeds disrespect for human life.  French translation: La guerre amène au mépris de la vie humaine.  Back-translation: War leads to the disregard of human life. | |
| 8. The desirable results of war have not received the attention they deserve.  French translation: Les résultats désirables de la guerre n’ont pas reçu l’attention qu’ils méritent.  Back-translation: The desirable results of war did not receive the attention they deserve. | |
| *Note.* Items were rated on a scale from 1 (Fortement en désaccord [Strongly disagree]) to 7 (Fortement d’accord [Strongly agree]). ^a^Items included in the shortened form of the scale. |  |
