# Supplementary material for: Are attitudes toward peace and war the two sides of the same coin? Evidence to the contrary from a French validation of the Attitudes Toward Peace and War Scale
Source: PLoS One. 2017 Sep 11;12(9):e0184001. doi: 10.1371/journal.pone.0184001 (PMC5593180; doi:10.1371/journal.pone.0184001)
Supplement: S2 File — (DOCX) [file pone.0184001.s002.docx]

| **S2 File.** | | | | | | | | |
| --- | --- | --- | --- | --- | --- | --- | --- | --- |
| EFA Pattern Loadings Based on Oblimin Rotation with Kaiser Normalization of Items Measuring Attitudes Toward Peace and War in Samples 1, 2, and 4. | | | | | | | | |
|  | Sample 1 | |  | Sample 2 | |  | Sample 4 | |
|  | F1 | F2 |  | F1 | F2 |  | F1 | F2 |
| **Peace subscale** |  |  |  |  |  |  |  |  |
| 1. | -**.75** | -.09 |  | -**.71** | -.03 |  | -**.56** | -.09 |
| 2. | **-.46** | -.03 |  | -.10 | -.26 |  | -.24 | -.06 |
| 3. | -**.79** | -.05 |  | -**.74** | -.05 |  | -**.73** | -.14 |
| 4. | -**.80** | -.08 |  | -**.60** | -.04 |  | -**.71** | -.05 |
| 5. | -**.55** | -.13 |  | -**.72** | -.02 |  | -**.53** | -.05 |
| 6. | -.35 | -.08 |  | -.33 | -.21 |  | -.35 | -.23 |
| 7. | **-.40** | -.04 |  | **-.51** | -.03 |  | **-.59** | -.02 |
| 8. | -.24 | -.25 |  | -.38 | -.03 |  | -.21 | -.19 |
| **War subscale** |  |  |  |  |  |  |  |  |
| 1. | -.05 | **-.57** |  | -.04 | **-.52** |  | -.10 | **-.63** |
| 2. | -.01 | -**.73** |  | -.06 | -**.77** |  | -.00 | -**.69** |
| 3. | -.09 | **-.51** |  | -.13 | -.31 |  | -.11 | -.39 |
| 4. | -.16 | -**.77** |  | -.18 | -**.72** |  | -.11 | -**.73** |
| 5. | -.03 | -**.77** |  | -.02 | -**.74** |  | -.05 | -**.74** |
| 6. | -.04 | -.38 |  | -.09 | -.36 |  | -.16 | -.30 |
| 7. | -.14 | -.37 |  | -.24 | -.37 |  | -.09 | -.29 |
| 8. | -.09 | -.20 |  | -.00 | -.29 |  | -.27 | -.13 |
| *Note.* F = factor. Absolute loadings ≥ .40 are shown in bold. | | | | | | | | |
