# Supplementary material for: Are attitudes toward peace and war the two sides of the same coin? Evidence to the contrary from a French validation of the Attitudes Toward Peace and War Scale
Source: PLoS One. 2017 Sep 11;12(9):e0184001. doi: 10.1371/journal.pone.0184001 (PMC5593180; doi:10.1371/journal.pone.0184001)
Supplement: S3 File — (DOCX) [file pone.0184001.s003.docx]

| **S3 File.** | | | | | | | | |
| --- | --- | --- | --- | --- | --- | --- | --- | --- |
| CFA Standardized Loadings of Items Measuring Attitudes Toward Peace and War in Samples 1, 2, and 4. | | | | | | | | |
|  | Sample 1 | |  | Sample 2 | |  | Sample 4 | |
|  | F1 | F2 |  | F1 | F2 |  | F1 | F2 |
| **Peace subscale** |  |  |  |  |  |  |  |  |
| 1. | -**.64** | -— |  | -**.70** | -— |  | -**.58** | -— |
| 2. | **-.52** | -— |  | -.24 | -— |  | -.27 | -— |
| 3. | -**.76** | -— |  | -**.72** | -— |  | -**.62** | -— |
| 4. | -**.77** | -— |  | -**.59** | -— |  | -**.69** | -— |
| 5. | -**.63** | -— |  | -**.74** | -— |  | -**.51** | -— |
| 6. | -.39 | -— |  | **-.44** | -— |  | **-.49** | -— |
| 7. | **-.41** | -— |  | **-.49** | -— |  | **-.61** | -— |
| 8. | -.38 | -— |  | -.35 | -— |  | -.31 | -— |
| **War subscale** |  |  |  |  |  |  |  |  |
| 1. | -— | **-.56** |  | -— | **-.51** |  | -— | **-.53** |
| 2. | -— | -**.78** |  | -— | -**.78** |  | -— | -**.72** |
| 3. | -— | **-.56** |  | -— | -.36 |  | -— | **-.44** |
| 4. | -— | -**.63** |  | -— | -**.60** |  | -— | -**.65** |
| 5. | -— | -**.72** |  | -— | -**.73** |  | -— | -**.81** |
| 6. | -— | -.35 |  | -— | -.36 |  | -— | -.38 |
| 7. | -— | **-.43** |  | -— | **-.45** |  | -— | -.28 |
| 8. | -— | -.25 |  | -— | -.28 |  | -— | -.30 |
| *Note.* F = factor. Absolute loadings ≥ .40 are shown in bold. | | | | | | | | |
