# Supplementary material for: Are attitudes toward peace and war the two sides of the same coin? Evidence to the contrary from a French validation of the Attitudes Toward Peace and War Scale
Source: PLoS One. 2017 Sep 11;12(9):e0184001. doi: 10.1371/journal.pone.0184001 (PMC5593180; doi:10.1371/journal.pone.0184001)
Supplement: S4 File — (DOCX) [file pone.0184001.s004.docx]

| **S4 File.** | | | | | | | | | |
| --- | --- | --- | --- | --- | --- | --- | --- | --- | --- |
| Semi-Partial Correlations Between Measures for the Shortened Form of the APWS in Samples 1, 2, and 4. | | | | | | | | | |
|  | Peace subscale | | | |  | War subscale | | | |
|  | S1 | S2 | S4 | S5 |  | S1 | S2 | S4 | S5 |
| Political orientation | -.18** | -.24** | -.18* | -.29* |  | .20** | .08 | .16* | .16 |
| Attachment | .14* |  |  |  |  | .25*** |  |  |  |
| Glorification | .19** |  |  |  |  | .19** |  |  |  |
| SDO |  | -.39*** |  | -.33** |  |  | .28*** |  | .33** |
| PD |  | .14 |  | .07 |  |  | -.05 |  | .05 |
| EC |  | .40*** |  | .32** |  |  | -.13 |  | -.07 |
| Willingness to fight for one’s country | .03 |  |  |  |  | .36*** |  |  |  |
| PBI |  | .56*** |  | .50*** |  |  | -.05 |  | -.17 |
| WBI |  | .07 |  | -.04 |  |  | .33*** |  | .16 |
| RWA |  |  |  | .10 |  |  |  |  | .41*** |
| SDR |  |  | .00 |  |  |  |  | .05 |  |
| *Note.* S = sample. SDO = social dominance orientation. PD = personal distress. EC = empathic concern. PBI = pro-peace behavioral intentions. WBI = pro-war behavioral intentions. RWA = right-wing authoritarianism. SDR = socially desirable responding. * *p* < .05. ** *p* < .01. *** *p* < .001. | | | | | | | | | |
