# Supplementary material for: Are attitudes toward peace and war the two sides of the same coin? Evidence to the contrary from a French validation of the Attitudes Toward Peace and War Scale
Source: PLoS One. 2017 Sep 11;12(9):e0184001. doi: 10.1371/journal.pone.0184001 (PMC5593180; doi:10.1371/journal.pone.0184001)
Supplement: S5 File — (DOCX) [file pone.0184001.s005.docx]

| **S5 File.** | | |
| --- | --- | --- |
| Mean Differences and Effects Sizes for Gender for the Shortened Form of the APWS in Samples 1 to 5. | | |
| Gender | Men | Women |
| APWS and samples | *η^2^* | *η^2^* |
| Peace subscale  Sample 1  Sample 2  Sample 3  Sample 4  Sample 5 | 5.45 (1.18)  .01***  4.95 (1.34)  .04***  5.28 (1.03)  .05**  5.74 (1.07)  .00***  4.98 (0.72)  .10*** | 5.71 (0.96)  5.44 (1.06)  5.71 (0.88)  5.71 (1.17)  5.67 (1.00) |
| War subscale  Sample 1  Sample 2  Sample 3  Sample 4  Sample 5 | 3.48 (1.44)  .09***  3.26 (1.27)  .07***  3.10 (1.18)  .13***  2.83 (1.26)  .02***  3.60 (0.96)  .07*** | 2.63 (1.14)  2.55 (1.15)  2.28 (0.95)  2.70 (1.33)  2.86 (1.29) |
| *Note.* Standard deviations are between brackets. Effect size was calculated using *η^2^* = eta squared. * *p* < .05. ** *p* < .01. *** *p* < .001. | | |
